# Fabrication and Characterization of Polylactic Acid/Hyaluronic Acid/Collagen Nanofibers for Tendon Tissue Engineering

**DOI:** 10.3390/polym18202475

**Published:** 2026-10-10

**Authors:** Zekiye Akdag, Cengiz Kazdal, Elif Nur Yildiz, Zehra Kanli, Ridvan Yildirim, Ali Sahin, Fatih Eren, Oguzhan Gunduz

**Affiliations:** 1 Department of Metallurgical and Materials Engineering, Graduate School of Science and Engineering, Marmara University, Istanbul 34854, Turkey; zekiyeakdag@marun.edu.tr; 2 Department of Orthopaedics and Traumatology, Faculty of Medicine, Recep Tayyip Erdogan University, Rize 53200, Turkey; 3 Department of Bioengineering, Faculty of Chemical and Metallurgical Engineering, Yildiz Technical University, Istanbul 34210, Turkey; nur.yildiz@yildiz.edu.tr; 4 Department of Biophysics, Faculty of Medicine, Istanbul Arel University, Istanbul 34010, Turkey; zehrakanli@arel.edu.tr; 5 Department of Metallurgical and Materials Engineering, Faculty of Technology, Marmara University, Istanbul 34854, Turkeyucemogu@ucl.ac.uk (O.G.); 6 Department of Biochemistry, School of Medicine, Genetic and Metabolic Diseases Research and Investigation Center, Marmara University, Istanbul 34854, Turkey; alisahin@marmara.edu.tr; 7 Department of Medical Biology, Faculty of Medicine, Recep Tayyip Erdogan University, Rize 53200, Turkey; fatih.eren@erdogan.edu.tr

**Keywords:** tendon tissue engineering, biomaterials, electrospinning, polylactic acid, collagen, hyaluronic acid, mesenchymal stem cells

## Abstract

Tendons are highly elastic connective tissues that transfer mechanical loads from muscle to bone, and their healing is often slow and incomplete because of limited vascularization. Tissue engineering approaches are therefore emerging as promising alternatives to support tendon repair. In this study, polylactic acid (PLA)-based nanofibers containing hyaluronic acid (HA) and collagen (COL), intrinsic components of the extracellular matrix, were fabricated by blend and coaxial electrospinning. Matrices of identical overall composition were produced by both configurations, so that the contribution of nanofiber architecture could be isolated from that of chemical composition; the coaxial nanofibers comprised a load-bearing PLA/COL core and a PLA/HA shell, reproducing the collagen-rich tendon proper and the hyaluronic acid-rich gliding surface of native tendon. Mean fiber diameters ranged from 319 ± 82 nm for neat PLA to 854 ± 220 nm for the coaxial nanofibers, approximately twice that of the compositionally identical blend nanofibers (454 ± 110 nm). Tensile strength increased from 2.108 MPa for neat PLA to 4.94 MPa for PLA/COL, and the coaxial nanofibers retained a comparable value (4.651 MPa, *p* > 0.05) despite the incorporation of HA. In human mesenchymal stem cells, viability was maintained or increased over 7 days on the coaxial scaffolds but declined markedly on the blend scaffolds, indicating that the core–shell architecture is more favorable for tendon tissue regeneration.

## 1. Introduction

Tendons are extremely elastic and strong connective tissues that link muscles to bones, enabling the transfer of mechanical loads. Injuries typically result from traumatic impacts, overuse, or degenerative diseases, and healing may be a slow and inadequate process due to limited blood circulation [1]. Conventional tendon repair methods include surgical interventions and autologous graft applications but suffer from biomechanical incompatibility, donor site damage, and inadequate tissue regeneration [2]. In recent years, tissue engineering-based strategies have emerged as promising alternatives to support tendon healing. These approaches aim to develop functional and biocompatible tendon tissues through the integration of biomaterials, cellular therapies, and biological signals.

Electrospinning is a novel technique widely used in the production of nanofiber-based biomaterials, offering a broad range of applications in the biomedical approach. This method enables the production of nanostructured materials through the conversion of polymer solutions or melts into fine fibers under high voltage. Nanofibers produced by electrospinning have a high surface area/volume ratio, a porous structure, and biomimetic properties that enhance cell–cell interactions. These properties enable their use in guiding cellular growth and mimicking the extracellular matrix (ECM) in regenerative medicine applications such as tendon tissue engineering (TTE). Electrospun nanofibers based on biocompatible polymers, such as poly-caprolactone (PCL), polylactic acid (PLA), and collagen (COL) are evaluated as potential biomaterials for tendon repair by promoting cell adhesion, proliferation, and differentiation [3,4].

PLA is a Food and Drug Administration (FDA)-approved biocompatible polymer and is used in a wide range of applications, from wound dressings to surgical sutures, bone fixation systems, and controlled drug delivery applications [5]. It has been demonstrated that PLA can be successfully electrospun using different solvent systems [6,7], and the resulting fibers can be modified with various functional components to impart specific properties for biomedical applications [8]. The high elasticity and tensile strength derived from its synthetic structure make PLA a suitable platform material for stable and reproducible fiber production [9]. Particularly in TTE applications, PLA’s mechanical durability and controlled degradation profile enable the creation of scaffolds that mimic the hierarchical structure of natural tendon tissue and contribute to tendon regeneration by supporting tenocyte proliferation and collagen production [10].

Hyaluronic acid (HA) is a glycosaminoglycan naturally found in the extracellular matrix (ECM). It is present in high concentrations in rooster combs, synovial fluid, the vitreous structure of the eye, and the umbilical cord. HA regulates tissue moisture balance through its hygroscopic properties, controlling regional hydration during the wound healing process and supporting cell proliferation and migration [11]. Ionescu et al. produced HA/polyethylene oxide-based electrospun nanofibers and performed their characterization and biological viability tests. As a result of their work, they demonstrated that HA increased the viability rates of cells [12]. In tendon tissue, HA reduces intra-tissue friction during mechanical loading by facilitating the movement of tenocytes and contributes to COL fibril orientation by regulating the organization of the tendon matrix. Furthermore, it may prevent excessive scar tissue formation by modulating inflammation during the tendon repair process [13].

COL is a natural polymer with a wide range of biomedical applications, from biosensors to tissue engineering. Despite its high biocompatibility, its insufficient mechanical strength limits its use in scaffold applications [14]. To overcome this limitation, COL is combined with synthetic polymers and used as an active component in hybrid biomaterial compositions [15]. Type I COL, the main structural component of tendon tissue, strengthens cell–matrix interactions by forming the natural microenvironment of tenocytes and supports tenocyte adhesion, proliferation, and tenogenic differentiation by containing cell-binding motifs such as RGD (Arg-Gly-Asp). The use of COL-containing scaffolds in tendon regeneration provides cells with a biomimetic environment, allowing the structural and biochemical properties of natural tendon ECM to be mimicked [16]. Several polymer systems compete for this role in TTE, and each carries a characteristic trade-off. PCL is FDA-approved and spins easily, but it is strongly hydrophobic, presents few cell-binding motifs and degrades over a period of years, which can outlast the healing window. PLGA and PGA reach higher initial strength and stiffness yet degrade quickly enough that mechanical support may be lost before the repair matures, and their acidic degradation products lower local pH [17]. Silk fibroin performs well mechanically and, when blended with PCL, raises expression of tendon markers such as type I COL and biglycan [18]; batch-to-batch variability and slow proteolytic degradation remain practical obstacles. Natural polymers used alone, COL among them, give the most faithful biochemical environment but are too weak to bear load [14]. PLA occupies the middle ground: it holds its strength longer than PLGA, degrades faster than PCL, and is considerably stiffer than any of the natural polymers, which is why it was selected here as the structural component.

Recent work has shifted away from the choice of polymer and toward how that polymer is organized. Nanofiber alignment is the clearest example, since aligned nanofibers direct cell orientation through contact guidance and raise tenogenic marker expression relative to random mats [18]. Others have built hierarchical macro-architectures, stacking or braiding aligned mats to close the gap with native tissue [19], or have loaded nanofibers with growth factors such as bFGF to drive tenogenic differentiation of stem cells [20]. One limitation is common to all of these approaches and is worth stating plainly: nonwoven electrospun scaffolds remain far weaker than the tissue they are meant to replace, and cell infiltration into dense nanofiber networks is poor [19]. The human patellar tendon reaches an ultimate tensile strength near 60 MPa, roughly an order of magnitude above what nonwoven mats typically achieve. Electrospun scaffolds are therefore better understood as temporary regenerative templates than as mechanical substitutes, and the design problem becomes how to present the right biochemical cues at the right location while the tissue rebuilds its own load-bearing capacity.

Electrospinning itself is a mature and widely applied fabrication route, and the novelty of the present work does not reside in the technique. The biological and mechanical performance of an electrospun scaffold is governed less by the spinning process than by the chemistry of its constituents and, critically, by how those constituents are distributed within the individual nanofiber. In blend electrospinning the components are randomly dispersed throughout the nanofiber cross-section, so that a hydrophilic ECM molecule such as HA is partly buried within the hydrophobic PLA matrix and partly exposed at the surface in an uncontrolled manner. Coaxial electrospinning, by contrast, allows the same components to be deliberately partitioned between a core and a shell.

This distinction is functionally meaningful for tendons. Native tendon is not compositionally uniform: a type I COL-rich tendon proper carries the tensile load; whereas, the surrounding epitenon, paratenon and tendon sheath are enriched in HA, which lubricates the gliding surface, limits fibroblast attachment, and thereby restricts peritendinous adhesion [21,22]. A PLA/COL core surrounded by a PLA/HA shell therefore reproduces this load-bearing-core/lubricating-surface organization at the level of the single nanofiber; whereas, a blend of the same three components cannot. PLA/COL blends mimicking tendon fascicles [23] and HA-containing core–sheath membranes for peritendinous applications [24,25] have each been reported; notably, in the latter HA was confined to the core and released as an anti-adhesion agent; whereas, here HA is positioned in the shell so that it is presented at the cell-contacting surface of a regenerative scaffold. To the best of our knowledge, HA and COL have not previously been combined within a single ternary PLA-based nanofiber in this tendon-mimetic core–shell arrangement. Furthermore, blend and coaxial architectures are rarely compared at an identical overall composition, since the two configurations are usually reported in separate studies with different polymers, solvents and processing windows, which makes the contribution of nanofiber architecture impossible to isolate from that of composition.

In this study, nanofibers containing PLA, HA, and COL were produced using both blend and coaxial electrospinning techniques for TTE applications. It is anticipated that the properties of the PLA-based nanofiber matrix will be enhanced through the incorporation of HA and/or COL, produced using different electrospinning configurations. The determination of the comparative effects of blend and coaxial electrospinning methods on nanofiber morphology, mechanical performance, thermal behavior, swelling/degradation behaviors, and cell–scaffold interactions constitutes the distinctive aspect of this research. The characterization of the nanofiber structures produced was performed using Fourier transform infrared spectroscopy (FT-IR), scanning electron microscopy (SEM), differential scanning calorimetry (DSC), and tensile tests. In vitro cytotoxicity, cell viability, and cell adhesion behaviors were evaluated in human mesenchymal stem cells (hMSCs) using the MTT assay and fluorescence microscopy. This multidisciplinary approach aims to contribute to the design of biomimetic and functional nanofibrous scaffolds with clinical translation potential for the regeneration of tendon injuries. The novelty of this study therefore lies in the biomimetic, spatially resolved design of a ternary PLA/HA/COL nanofiber and in the direct, composition-matched comparison of blend and coaxial architectures, rather than in the electrospinning process itself.

## 2. Materials and Methods

### 2.1. Materials

Poly (lactic acid) (PLA) 4060D (MW~92 kDa) as powdered form was purchased from Plymouth, MN, USA. COL type I was obtained from Collagen R&D company, Istanbul, Turkey. Chloroform is purchased from Merck KGaA, Darmstadt, Germany. Hyaluronic acid was obtained from Amhwa Biopharm Co., Ltd., Binzhou, Shandong, China. Phosphate-buffered saline (PBS, pH = 7.4) and Tween-80 (surfactant) were purchased from Sigma-Aldrich, Poole, UK.

### 2.2. Production of Nanofibers

Nanofibers were produced via the electrospinning (NS24, Inovenso Co., Istanbul, Turkiye) method using solutions containing PLA, COL, and HA (Table 1). Initially, 10% PLA was dissolved in chloroform about 2 h by using a magnetic stirrer. Then, in accordance with the experimental groups outlined in table, 1% COL and/or HA aqueous form were added to the 10% PLA solution and thoroughly mixed 30 min at room temperature with a magnetic stirrer. Finally, Tween 80 was incorporated as a surfactant, and the solutions were prepared for nanofiber production.

During the electrospinning process, optimal fiber structure was achieved by setting the voltage in the range of 27–29 kV, the flow rate between 0.5 and 1.25 mL/h, the needle-to-collector (rotating drum collector) distance at 12 cm vertically, in which the spinneret was mounted above the collector and the jet traveled upwards, and the average processing time at 4 h by using 19G needle at (22 ± 2) °C and (45 ± 5)% relative humidity. The operating parameters were optimized to ensure the fibers exhibited uniform morphological characteristics and the homogeneous distribution of components.

### 2.3. Characterizations of Nanofibers

#### 2.3.1. Chemical Analysis of the Nanofibers

Fourier transform infrared spectroscopy (Jasco, FT-IR 4700, Pfungstadt, Germany) was used to investigate the chemical structures of nanofibers. The measurements were performed at room temperature between 4000 and 400 cm^−1^ wavelengths with 32 scans averaged at 4 cm^−1^ resolution.

#### 2.3.2. Morphological Analysis of the Nanofibers

Scanning electron microscopy (EVO MA 10, Zeiss, San Diego, CA, USA) was utilized to examine the morphology of the electrospun nanofibers. Before taking the SEM image, the surface of the nanofibers cut to size was coated with a thin layer of gold using a sputter coater (SC7620, Quorum, Lewes, UK) for 120 s. A voltage of 10 kV was applied for analysis, and a range of magnifications were employed for scans. Following the acquisition of SEM images, 100 fibers were randomly selected, and their diameters were determined by means of imaging software analysis (Olympus AnalySIS version 2.1.2, Tokyo, Japan). This enabled the performance of a dimensional analysis for each sample.

#### 2.3.3. Thermal Analysis of the Nanofibers

Differential scanning calorimetry (DSC) (DSC-60 Plus, Shimadzu, Tokyo, Japan) was used to examine and interpret the produced electrospun fibers in terms of their thermal properties, such as melting temperature (T_m_) and glass transition temperature (T_g_). To obtain the results, the samples were exposed to a heating rate of 10 °C/min, and the measurements were analyzed in the range of 25 °C to 350 °C. The measurements were carried out in a static air atmosphere; no inert purge gas was applied, and the gas flow rate was therefore not controlled.

#### 2.3.4. Mechanical Analysis of the Nanofibers

The mechanical behavior of the electrospun nanofibers was evaluated using a vertical axis tensile testing instrument (EZ-LX, Shimadzu, Tokyo, Japan). Prior to conducting the tests according to established standards. Each sample exhibited an average width of 10 mm and a height of 50 mm. The width of all samples was accurately measured and documented utilizing a digital micrometer (Mitutoyo MTI Corp., New York, NY, USA). The tensile tests were performed by applying a pulling force on each fiber sample at a controlled speed of 5 mm/min.

#### 2.3.5. Swelling and Degradation Analysis of the Nanofibers

The swelling and degradation characteristics of the nanofibers were assessed. The assessment of the swelling behavior of electrospun nanofibers was conducted by placing the samples in 1 mL of PBS at a pH of 7.4 and incubating them at 37 °C on a thermal shaker (BIOSAN TS-100, Riga, Latvia) [26]. On the first day, the dry weight ($W_{0}$) of the nanofibers was determined. On the following days (1–7th days), the wet weight ($W_{w}$) of the nanofibers was measured, and the swelling ratio (S) was calculated using Equation (1).

(1) $$S=\frac{W_{w}-W_{0}}{W_{0}}\times 100,$$

The degradation behavior of the electrospun nanofibers was evaluated by placing equal weight ($W_{i}$) samples in 1 mL of PBS at a pH of 7.4. The samples were then incubated at 37 °C on a thermal shaker. Thereafter, the samples were separated from their liquids and dried at 37 °C. The degradation value (*D*) was calculated using Equation (2), where ($W_{i}$) is the initial weight and ($W_{f}$) is the final weight.

(2) $$D=\frac{W_{i}-W_{f}}{W_{i}}\times 100,$$

#### 2.3.6. Cell Culture Studies

The viability of human mesenchymal stem cells (hMSCs, ATCC, Rockville, MD, USA) on nanofibers was quantitatively determined by the MTT (3-(4,5-Dimethylthiazol-2-yl)-2,5-diphenyltetrazolium bromide) test (1 × 10^4^ cells per well) for 1, 3, and 7 days. After incubation (37 °C, 5% CO_2_), the culture medium was discarded, and the nanofibers were washed three times with PBS solution. A volume of 90 µL fresh medium and 10 µL MTT solution (5 mg mL^−1^ in PBS solution) were added into the 96 well-plate and incubated at 37 °C, in a 5% CO_2_ atmosphere for 3 h. Following this, the samples were transferred to clean plates. An amount of 200 µL of DMSO was added to dissolve the formazan crystals and the resulting suspension was incubated for 10 min. Finally, the measurement of the absorbance values was conducted at 540 nm with a microplate reader (Multiskan SkyHigh, Thermo Fisher Scientific, Waltham, MA, USA).

Cell adhesion and proliferation were visualized using fluorescence microscopy. Nanofibers were collected on days 1, 3, and 7 after culture and washed twice with PBS solution. The cells were fixed with 4% paraformaldehyde for 30 min, then stained with DAPI (4′,6-diamidino-2-phenylindole) solution and kept in the dark for 10 min. Images were captured using a fluorescence microscope at 20× magnification.

For SEM analysis, the culture medium was removed from the plate, and all nanofibers were fixed with 4% glutaraldehyde. Then, the nanofibers were dehydrated with 70% diluted ethanol and dried at room temperature. Prior to SEM analysis, the dried nanofibers were coated with gold/palladium (Au/Pd) for 60 s. Nanofibers were examined using SEM.

#### 2.3.7. Statistical Analysis

The Standard Error of the Mean (±SEM) of three or more experiments is used to express the data. Tukey’s post-tests were used after the two-way variance analysis to determine all statistical analyses. Tukey’s multiple comparisons test was used for all statistical tests, which were carried out using GraphPad Software Inc.’s Prism 8.0.2 version (Boston, MA, USA).

## 3. Results

### 3.1. FT-IR Analysis of the Nanofibers

FT-IR analysis is used to show the chemical composition of the produced nanofiber structures and the interactions between components (Figure 1). In the spectrum of pure PLA nanofibers, the characteristic peaks of PLA are at 1749 cm^−1^ for the carbonyl (C=O) stretching vibration, CH_3_ deformation vibrations at 1452 cm^−1^ and 1382–1359 cm^−1^, and C-O-C ester bond stretching vibrations in the 1265–1180 cm^−1^ range [27]. In composite nanofiber structures (10% PLA, 1% COL, 1% HA), the spectra predominantly show PLA characteristics because PLA has the highest concentration of these nanofibers. However, in nanofibers containing PLA/COL and PLA/HA/COL, slight shoulders corresponding to the amide I and amide II bands are observed in the 1632–1537 cm^−1^ region, indicating that COL has been successfully incorporated into the nanofiber structure. The slight shift (from 1749 cm^−1^ to 1743–1751 cm^−1^) and broadening observed in the carbonyl peak of PLA are thought to be due to hydrogen bond interactions between PLA and COL and HA [28]. Due to the low concentration of HA (1%), the characteristic carboxylate (COO^−^) and hydroxyl group bands are masked by the strong peaks of PLA; however, the slight broadening and increase in intensity of the bands in the 1000–1200 cm^−1^ region indicate the presence of HA. The spectral changes observed in all composite structures confirm the presence of molecular-level interactions between the components.

### 3.2. SEM Analysis of the Nanofibers

SEM images and fiber diameter distributions of nanofibers were presented in Figure 2. The morphology and diameter thickness of wound dressings are critical for TTE. The measured average fiber diameters of PLA, PLA/COL, PLA/HA, PLA/HA/COL blend, and PLA/HA/COL coaxial nanofibers were 319 ± 82 nm, 582 ± 120 nm, 343 ± 91 nm, 454 ± 110 nm, and 854 ± 220 nm, respectively. Pure PLA nanofibers with the smallest fiber diameter exhibit a topological approach suitable for COL fibrils in the natural structure of tendon tissue (approximately 200–480 nm) [12,29]. The significant increase in diameter in the PLA/COL nanofibers indicates that the COL addition modulates the morphology through viscosity and surface tension during the electrospinning process. In the PLA/HA nanofibers, fibers with a more controlled and narrower diameter range were obtained due to HA; the effects of HA on cellular migration and tissue integration are also mentioned in the literature [30]. The PLA/HA/COL blend nanofibers increased heterogeneity in the diameter distribution with its combination of both COL and HA; this may be important for tendon-like stepwise structure designs. The coaxial nanofibers exhibited the largest mean diameter (854 ± 220 nm), approximately twice that of the compositionally identical blend nanofibers. This increase is an expected consequence of the coaxial configuration, in which a compound jet is formed from two separately fed solutions, and is itself consistent with the formation of a core–shell structure. Rather than enhancing tensile strength, which remained statistically indistinguishable from that of PLA/COL (Section 3.4), the larger diameter is advantageous for two reasons. First, a distinct spatial partitioning into a load-bearing PLA/COL core and a surface-presenting PLA/HA shell is not geometrically feasible at the 300–500 nm scale of the single-needle groups, so the increase in diameter is a prerequisite for the tendon-mimetic architecture targeted here. Second, diameters in this range have been reported to favor tenogenic differentiation and to attenuate the inflammatory response relative to finer nanofibers [17], while the associated increase in inter-nanofiber pore size may partially relieve the poor cell infiltration that limits dense nanofibrous mats [19]. The trade-off is a reduced surface-area-to-volume ratio and a departure from the 200–480 nm range of native collagen fibrils, which is why the coaxial nanofibers are best regarded as complementary to, rather than uniformly superior to, the finer single-needle groups.

For TTE applications, fiber diameter emerges as a critical parameter that directly influences the biomechanical performance and cellular behavior of tissues. Khatib et al. (2020) demonstrated that PLGA-based ultrafine fibers (1.27 µm) optimize tenogenic differentiation and the regulation of anti-inflammatory responses compared to finer fibers [17]. Accordingly, the ultra-fine fiber diameters obtained in the pure PLA and PLA/HA nanofibers offer a mechanically and biologically advantageous starting point.

### 3.3. DSC Analysis of the Nanofibers

DSC was employed here to determine the physical state of the nanofibers at physiological temperature rather than to evaluate their melting behavior as such, since the glass transition establishes whether the scaffold remains dimensionally stable at 37 °C. As seen in Figure 3, the glass transition temperature (Tg) of pure PLA was found to be approximately 60 °C and the melting temperature was between 150 and 155 °C [31]. In the PLA/HA nanofibers, Tg decreased to approximately 58 °C, while it was determined as 56–57 °C in the PLA/COL and around 55 °C in the PLA/HA/COL blend nanofibers. The lowest Tg was measured in the PLA/HA/COL coaxial nanofibers (54–55 °C). This decrease was associated with the increased mobility of the polymer chains in the presence of HA and COL. The melting temperature, in contrast, remained essentially unchanged in all groups, which is consistent with PLA constituting approximately 83 wt% of every composition. All formulations therefore remained well within the glassy state at physiological temperature. The DSC data are accordingly reported as supporting evidence for the physical state of the nanofibers rather than as a means of distinguishing between the formulations, and the degree of crystallinity was not quantified in this study.

### 3.4. Tensile Testing of the Nanofibers

The tensile properties of these nanofibers provide valuable insights into their suitability for TTE applications, where specific mechanical requirements are crucial. According to the Table 2, the pure PLA nanofibers demonstrate moderate strength (2.108 MPa) and high flexibility (84.713% strain), making them suitable for flexible structures. However, its lower strength may limit its application in load-bearing tendon repairs. By adding HA to PLA, the tensile strength increases to 3.425 MPa, though flexibility decreases. The increased stiffness can provide structural support in tendon repair, though it only partially replicates the natural flexibility of a healthy tendon [32]. The PLA/COL nanofibers achieve the highest tensile strength (4.94 MPa) and moderate flexibility (35.76%), providing a favorable balance of support and elasticity among the compositions tested, although still below the strength of native tendon [10]. COL improves both durability and elasticity, making this scaffold well-suited for replicating the properties of natural tendon tissue [33]. The PLA/HA/COL blend and coaxial nanofibers likewise offered balanced properties. Notably, the tensile strength of the coaxial configuration (4.651 MPa) was comparable to that of the PLA/COL group (4.94 MPa), with no statistically significant difference between them (*p* > 0.05), indicating that the core–shell architecture preserves mechanical integrity rather than compromising it. Combined with its limited strain, this strength profile is consistent with the durability requirements of tendon-like structures. The distinct advantage of the coaxial design therefore lies not in its mechanical performance but in its biological response, as the surface localization of COL and HA in the shell layer improved cytocompatibility (Figure 5). In summary, these findings indicate that adjusting the composition can fine-tune mechanical properties for TTE, creating an ideal balance of strength and flexibility to closely replicate the behavior of natural tendons.

### 3.5. Swelling and Degradation Properties of the Nanofibers

The swelling test results of electrospun nanofibers provide insight into the water retention capacity and structural integrity of nanofibers (Figure 4A). Pure PLA nanofibers exhibited a swelling rate of 441.7% on the 1st day, which decreased to 254.5% by the end of the 7th day; this indicates that PLA initially retains water to a limited extent due to its hydrophobic nature and subsequently releases it in a controlled manner over time. PLA/HA nanofibers initially exhibited a high-water uptake of 386.3%, but declined to 175.1% after 7 days, showing the sharpest decrease. This situation may be due to the rapid decline in structural stability resulting from the high hydrophilicity of HA. The highest initial swelling ratio was observed in the PLA/COL nanofiber group (513.2%), demonstrating that COL can strongly bind water due to its amido and hydroxyl groups. However, this value decreased to 244.0% over time and may be associated with relaxation of COL nanofibers. In contrast, the PLA/HA/COL blend nanofibers exhibited moderate swelling of 444.6% on the 1st day, decreasing to 258.5% on the 7th day, having a more stable profile. Water retention capacity and dimensional stability can be attributed to the combined structural stability of PLA and the hydrophilic nature of HA and COL. The PLA/HA/COL coaxial nanofibers exhibited the most controlled swelling behavior. The swelling rate, which was 419.7% on the 1st day, remained at 230.9% on the 7th day, showing the lowest rate of change. This behavior can be explained by the outer PLA/HA layer controlling water diffusion and the inner PLA/COL layer maintaining its mechanical integrity.

The degradation rates of electrospun PLA-based nanofibers varied depending on the composition (Figure 4B). The degradation rate of the pure PLA nanofibers was between 3.2% and 4.6%, with slow and steady weight loss occurring due to the hydrophobic structure of PLA. This property provides an advantage in applications requiring long-term mechanical strength, such as tendon tissue [34]. In the PLA/HA nanofibers group, degradation increased on the 2nd day (around 4.5%) due to the high hydrophilicity of HA and then followed a fluctuating behavior. On the other hand, the PLA/COL nanofibers group exhibited a more stable structure in the range of 2.6–3.9%, with the bonds formed between COL and PLA increasing matrix stability [10]. The highest degradation (4.4–5.7%) was observed in PLA/HA/COL blend nanofibers. The hydrophilic HA and the biodegradable COL may have accelerated the breakdown of the polymer matrix. In contrast, PLA/HA/COL coaxial nanofibers exhibited moderate degradation in the range of 3.9–5.1%; the PLA/HA outer layer increased water uptake, while the inner PLA/COL layer maintained structural integrity.

Accordingly, PLA/HA/COL blend nanofibers exhibited the highest degradation rate, while the pure PLA group showed the lowest. The PLA/HA/COL coaxial group exhibited a gradual and consistent mass loss throughout the incubation period, with the degradation profiles of all groups following a similar overall trend. These results suggest that coaxial structure is the most promising candidate for TTE in terms of both mechanical strength and time-dependent biodegradability.

### 3.6. In Vitro Cell Viability Results

Figure 5 presents the results of a cell viability assay conducted to evaluate the biocompatibility of different nanofiber compositions with hMSCs. Cell viability was assessed using the MTT assay on days 1, 3, and 7. On the first day, the PLA and PLA/COL groups exhibited approximately 65–70% viability, while the PLA/HA and PLA/HA/COL coaxial nanofibers showed higher cell viability. In contrast, the PLA/HA/COL blend group displayed lower cell viability compared to the other groups. By the third day, cell viability was maintained in the PLA and PLA/COL nanofibers; whereas, the PLA/HA nanofibers reached the highest viability level. However, a significant decrease in cell viability was observed in the PLA/HA/COL blend group. By the seventh day, cell viability remained stable or increased in the PLA, PLA/COL, and PLA/HA/COL coaxial groups. The PLA/HA group maintained the highest cell viability, while the PLA/HA/COL blend group exhibited a notable decrease.

Overall, the results suggest that the PLA/HA and PLA/HA/COL coaxial groups are biocompatible with hMSCs and support cell proliferation. However, given the observed reduction in cell viability, the PLA/HA/COL blend composition appears to be less suitable for hMSCs.

It should be noted that the observed cell viability percentages (ranging from ~30% to 80%) across the scaffold groups in 2D MTT assays can be attributed to the intrinsic physical and surface properties of the hydrophobic PLA matrix, as well as the spatial distribution of cells within a 3D nanofibrous architecture. In standard 2D culture settings, cells seeded onto 3D porous scaffolds face an initial adaptation period to the polymer surface energy and scaffold topography, which can reduce the apparent metabolic activity per well compared to flat tissue culture polystyrene controls. However, this response does not indicate acute chemical toxicity. As verified by fluorescence and SEM imaging (Figure 6 and Figure 7), hMSCs actively adhered to the pseudopodia, and proliferated over the 7-day period. In particular, the PLA/HA/COL coaxial nanofibers provided a highly supportive microenvironment, mitigating growth inhibition through the localized presence of biological ECM cues (HA and COL). For future vivo and invasive applications, surface modification techniques (e.g., plasma treatment or hydrophilic coating) or pre-conditioning procedures could be utilized to further enhance initial cell seeding efficiency and overcome surface-induced growth lag.

Fluorescence microscopy images and MTT assay results indicate that cell viability follows similar trends across different nanofiber compositions. The images reveal that cell adhesion and distribution vary depending on the nanofiber type, with PLA/HA/COL coaxial and PLA/HA nanofibers demonstrating better cell retention over time. The MTT assay confirms this observation, showing higher cell viability rates in these groups, particularly on days 3 and 7. In contrast, the PLA/HA/COL blend group exhibited the lowest cell viability. These findings suggest that PLA/HA and PLA/HA/COL coaxial nanofibers provide a more favorable microenvironment for cell attachment and survival (Figure 6).

SEM analyses clearly demonstrate the time-dependent adhesion and morphological changes in hMSCs on PLA, PLA/HA, PLA/COL, PLA/HA/COL blend, and PLA/HA/COL coaxial nanofiber composites (Figure 7). On the first day, most cells are spherical or slightly elongated and exhibit limited adhesion to the surface. Compared to the PLA nanofibers, it is noteworthy that cells in the PLA/HA and PLA/COL groups encounter the nanofibers over a larger surface area. The contributions of COL and HA, which are natural ECM components, to cell adhesion have been clearly demonstrated in the literature. COL enhances adhesion, survival, and proliferation in hMSCs [35]; HA functions as a hydrated matrix that facilitates attachment and supports cell migration in hMSCs [36,37]. On the fourth day, it was observed that the cells had significantly elongated, particularly in the PLA/HA and PLA/COL groups, tightly adhering to the nanofiber network with pseudopodia extensions and increased cell-nanofiber interaction. In PLA/HA/COL blend and PLA/HA/COL coaxial nanofibers, cells exhibited three-dimensional spreading not only on the surface but also between nanofibers, strengthening their integration into the nanofibers.

By the seventh day, cells exhibited marked proliferation in all composites, with nanofibers largely covered by cells, particularly in the PLA/HA/COL blend and PLA/HA/COL coaxial nanofibers, accompanied by intense cell–nanofiber interaction. This situation demonstrates that COL and HA additives support cell adhesion and proliferation. It is understood that cell morphology shows a more limited spread in the PLA control group; whereas biomimetic additives significantly promote cell growth. Tenogenic differentiation was not assessed in this study, and the expression of markers such as scleraxis and tenomodulin will be required to establish tenogenic potential. The nanofibers were also collected as randomly oriented mats rather than aligned architecture and were not subjected to dynamic mechanical stimulation. The coaxial scaffolds are therefore presented here as a regenerative template whose architecture merits further evaluation, and their suitability for tendon repair will require tenogenic differentiation studies and in vivo assessment in a tendon defect model.

## 4. Conclusions

In this study, nanofiber structures were successfully developed by adding HA and COL at different ratios to the PLA matrix using blend and coaxial electrospinning techniques for tendon tissue regeneration. PLA is chosen due to its high mechanical strength and FDA-approved biocompatibility properties; HA and COL are added to the formulation to enhance bioactivity and strengthen cell–material interaction as they exist in the ECM of native tendon structures. It has been determined that PLA/HA/COL nanofibers produced in a coaxial structure yield more successful results in terms of mechanical performance, cell viability, and cellular adhesion compared to the blend nanofiber group.

The results of FT-IR, SEM, DSC analysis, and tensile tests revealed that the incorporation of HA and COL into the composition caused significant changes in the physicochemical and mechanical properties of the nanofiber structures. Biocompatibility tests conducted using hMSCs demonstrated that none of the groups exhibited cytotoxicity, but the PLA/HA/COL scaffolds produced by coaxial electrospinning supported cell proliferation and viability at the highest level. The findings indicated that the coaxial electrospinning method is more advantageous than the blend method in the production of tendon tissue scaffolds. In conclusion, the combination of HA and COL with PLA has significantly improved the biological and mechanical performance of nanofiber structures, with coaxial structures emerging as a promising scaffold design that warrants further evaluation for TTE applications. Since the blend and coaxial groups were fabricated at an identical overall composition, the differences observed between them are attributable to nanofiber architecture rather than to chemistry, and they indicate that the spatial placement of HA and COL within the nanofiber determines scaffold performance. Perhaps increasing the COL content in the inner layer of these nanofibers can further improve mechanical properties and cell viability.

## Figures and Tables

**Figure 1:**
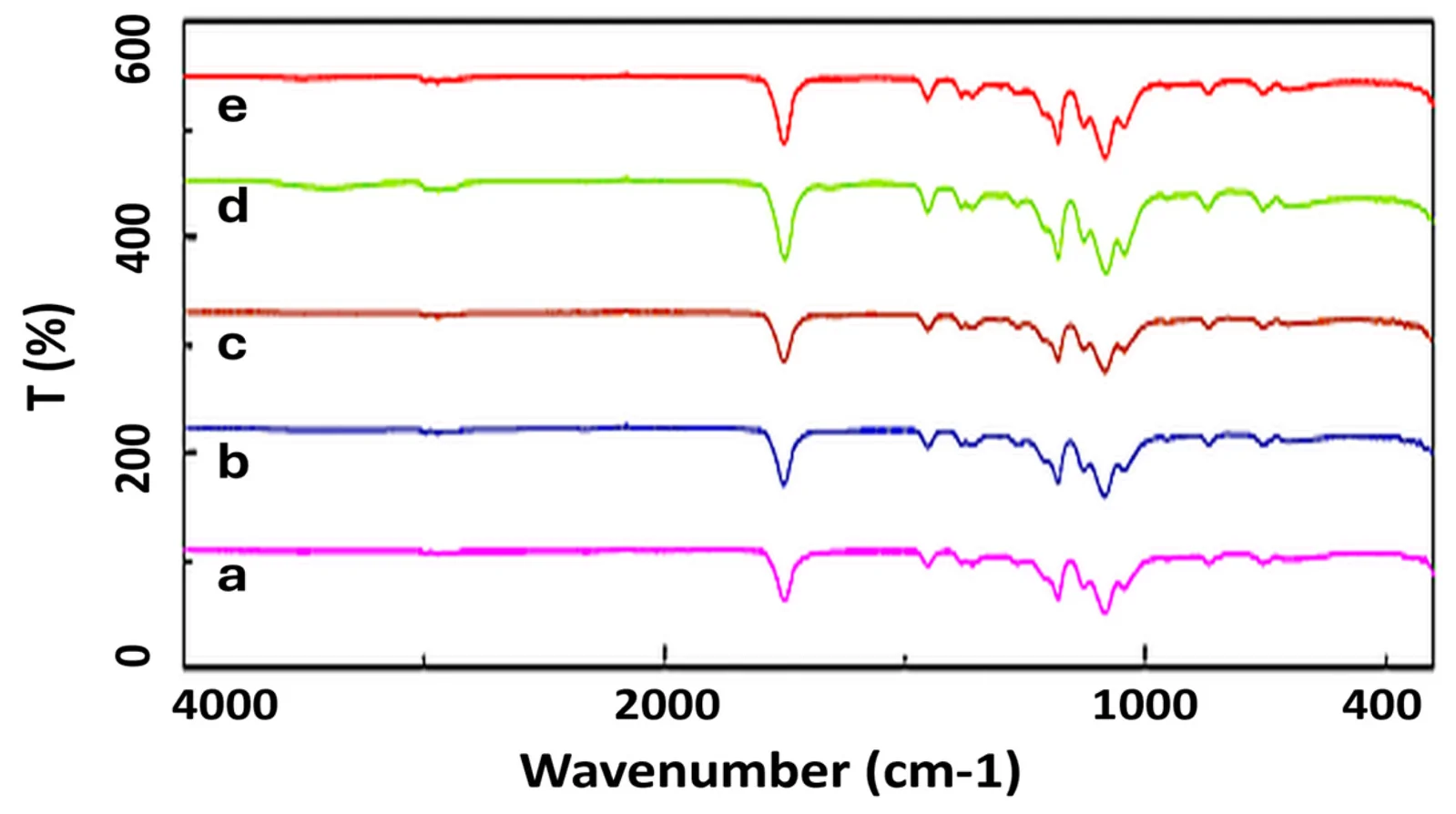
FT-IR spectrums of the (a) PLA, (b) PLA/COL, (c) PLA/HA, (d) PLA/HA/COL blend, and (e) PLA/HA/COL coaxial nanofibers.

**Figure 2:**
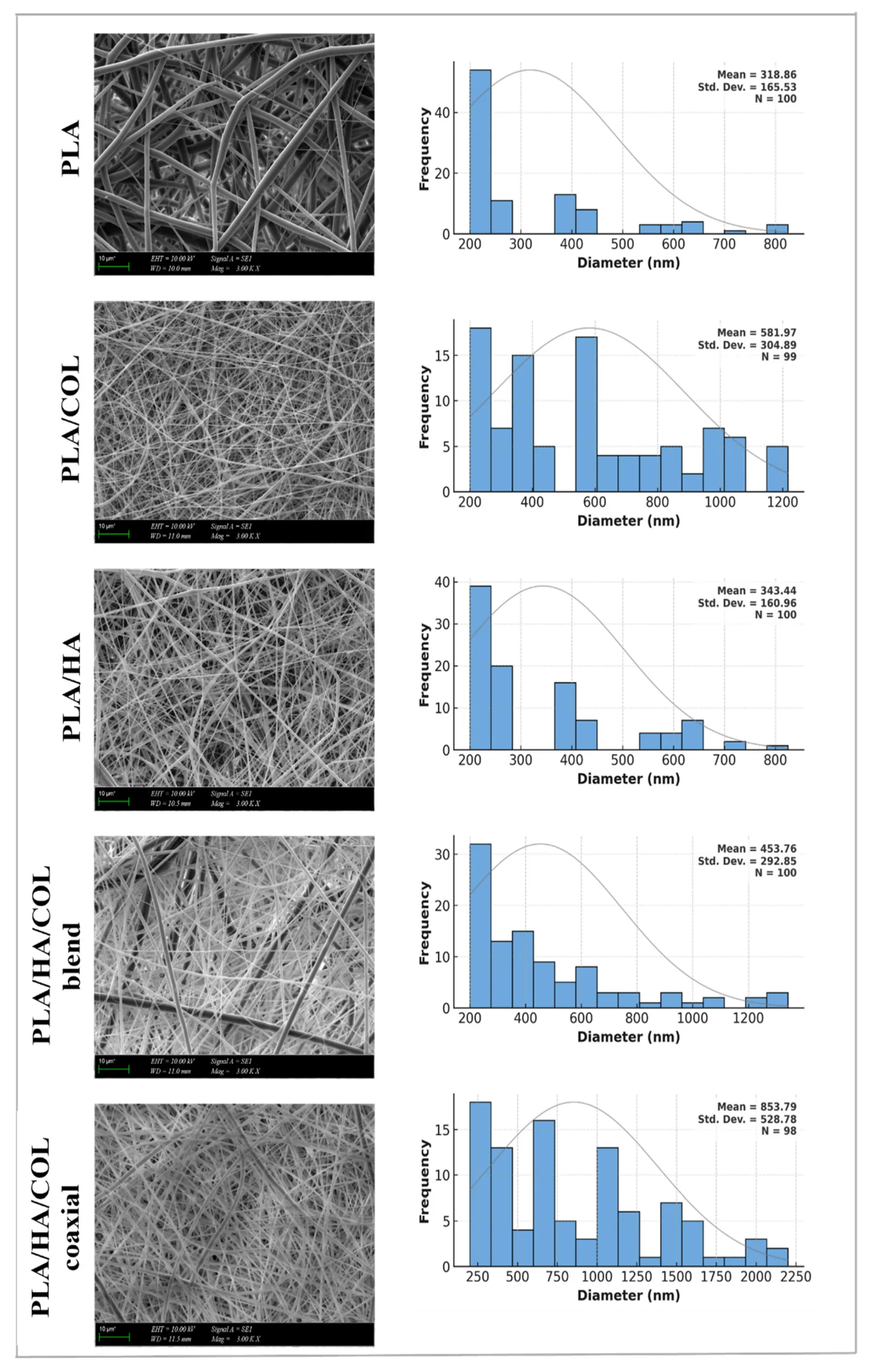
SEM images of the PLA, PLA/COL, PLA/HA, PLA/HA/COL blend, and PLA/HA/COL coaxial nanofibers.

**Figure 3:**
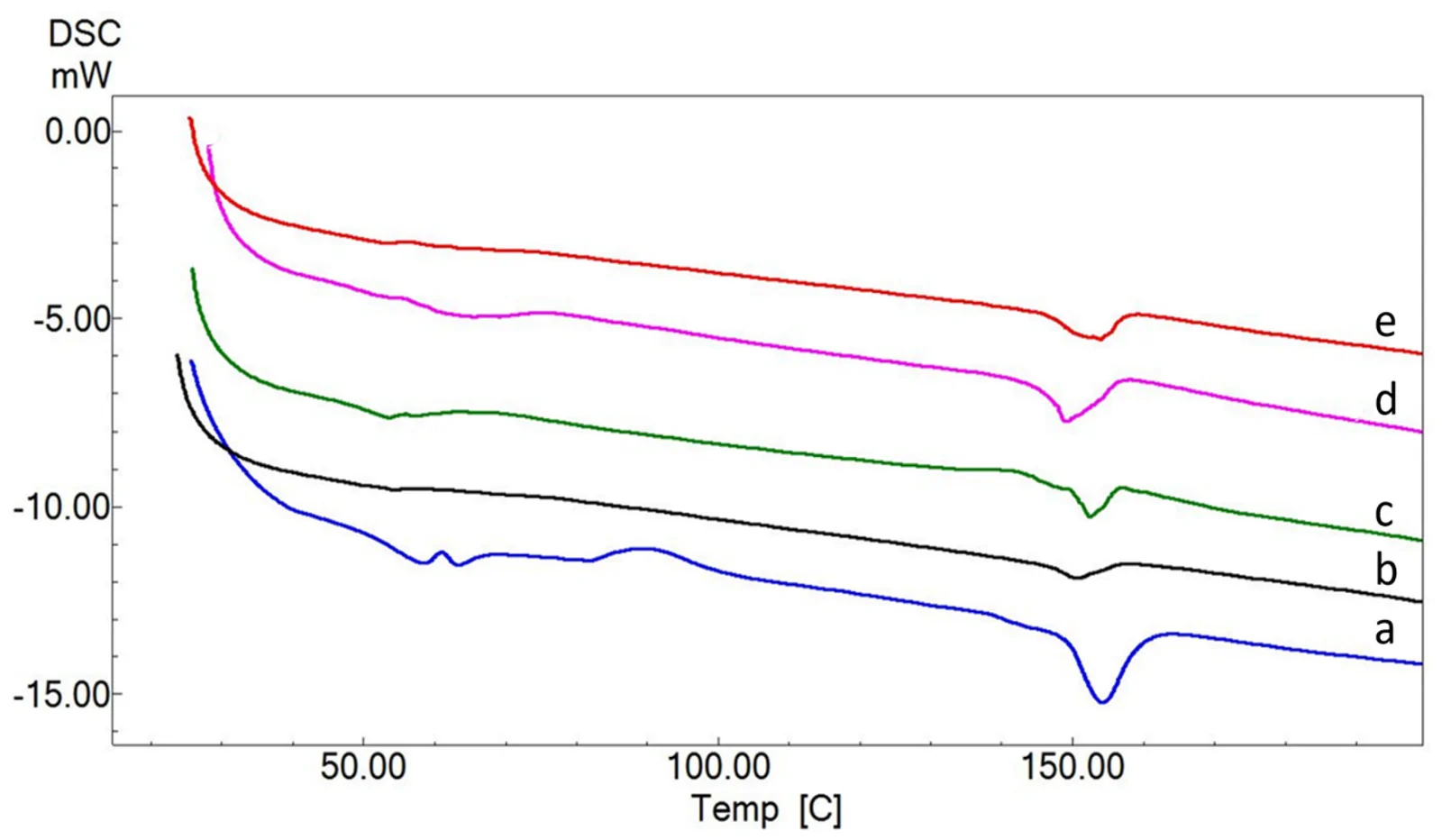
DSC curves of the nanofibers, (a) PLA, (b) PLA/COL, (c) PLA/HA, (d) PLA/HA/COL blend, and (e) PLA/HA/COL coaxial.

**Figure 4:**
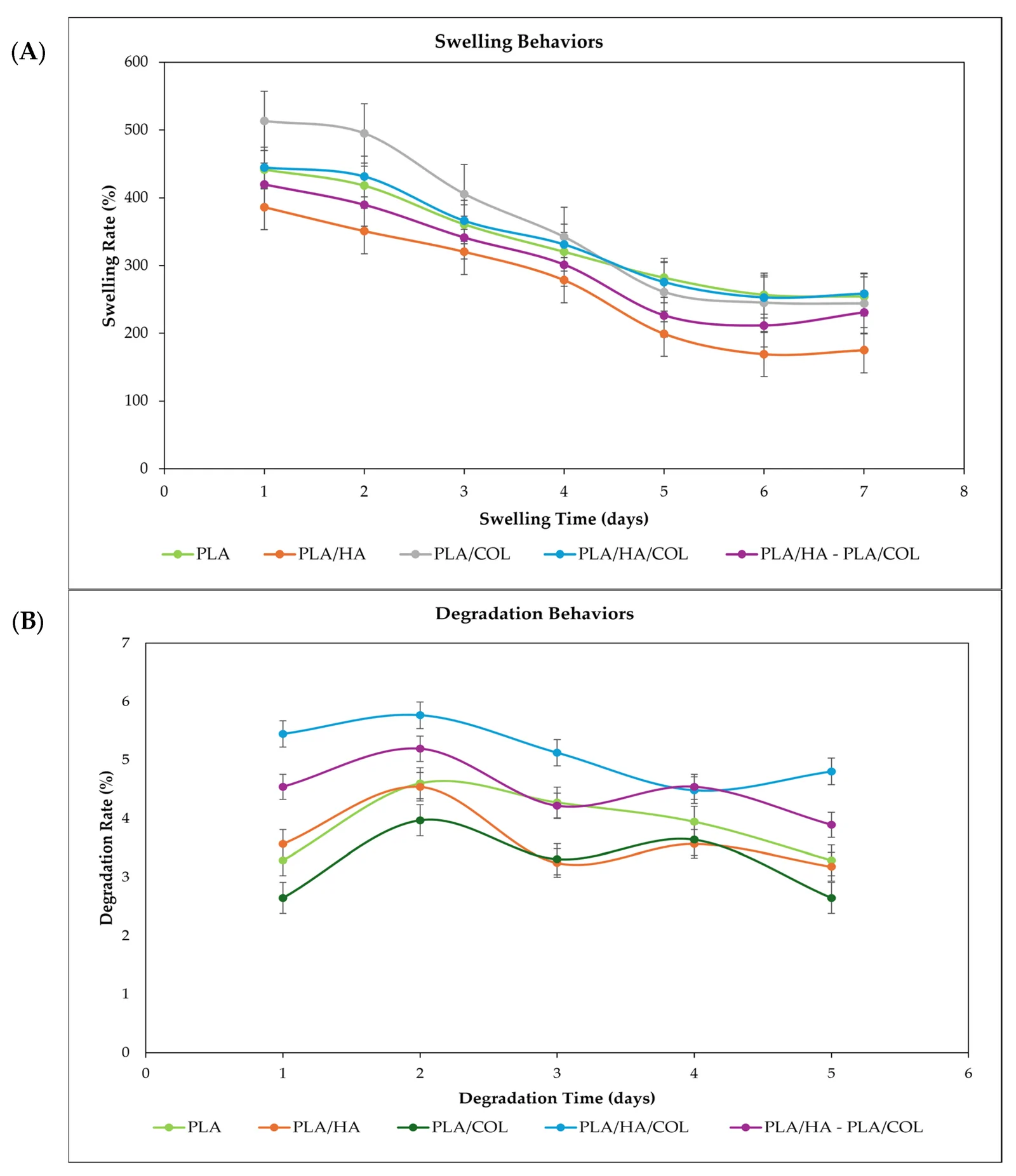
Swelling (**A**) and degradation (**B**) behaviors of nanofibers.

**Figure 5:**
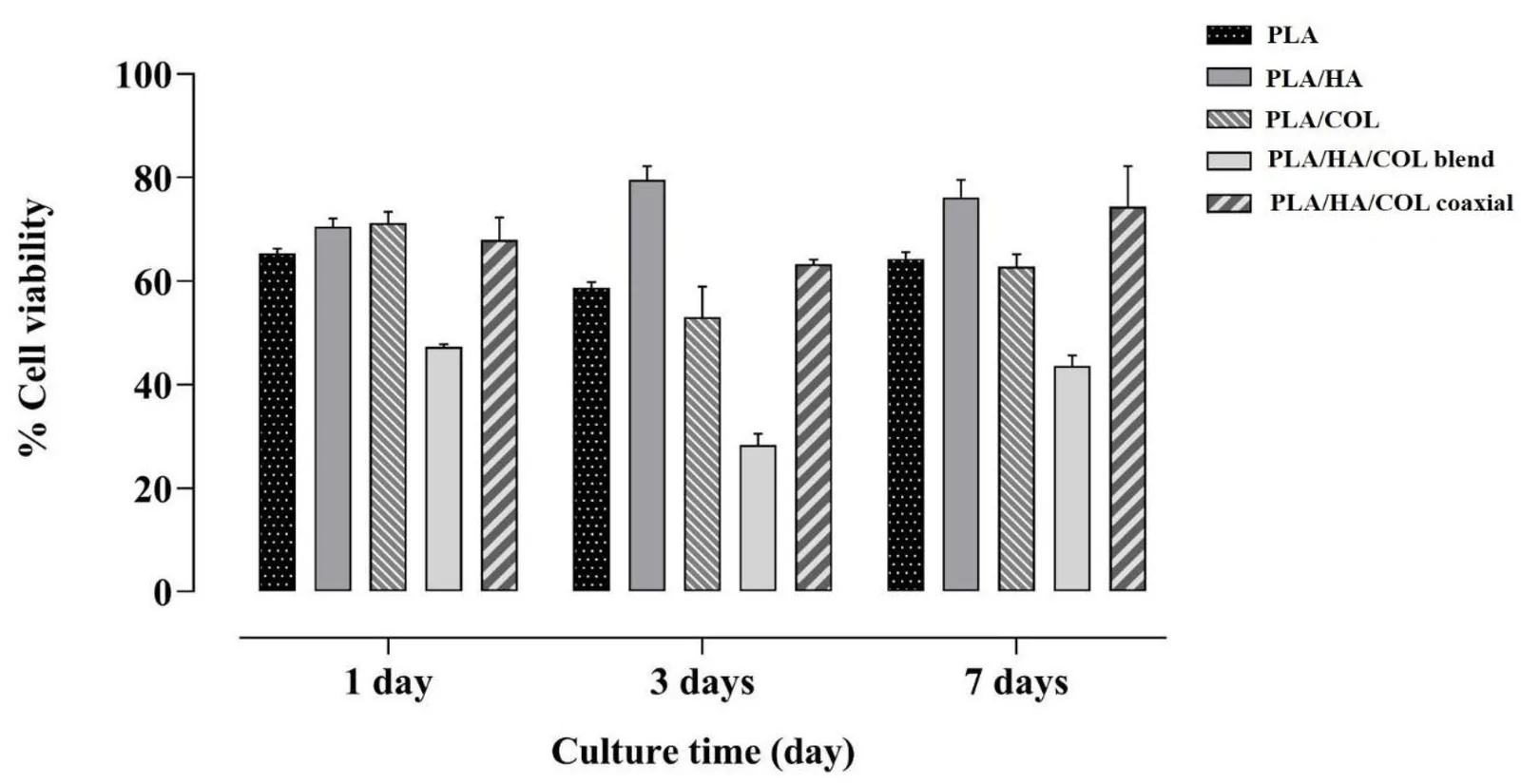
Cell viability of hMSCs cells was assessed for cytotoxicity using the MTT assay. The biocompatibility of hMSCs cells incubated with nanofibers was evaluated on days 1, 3, and 7. All experiments were performed in triplicate, and the results are expressed as mean ± SEM (standard error of the mean) (n = 3).

**Figure 6:**
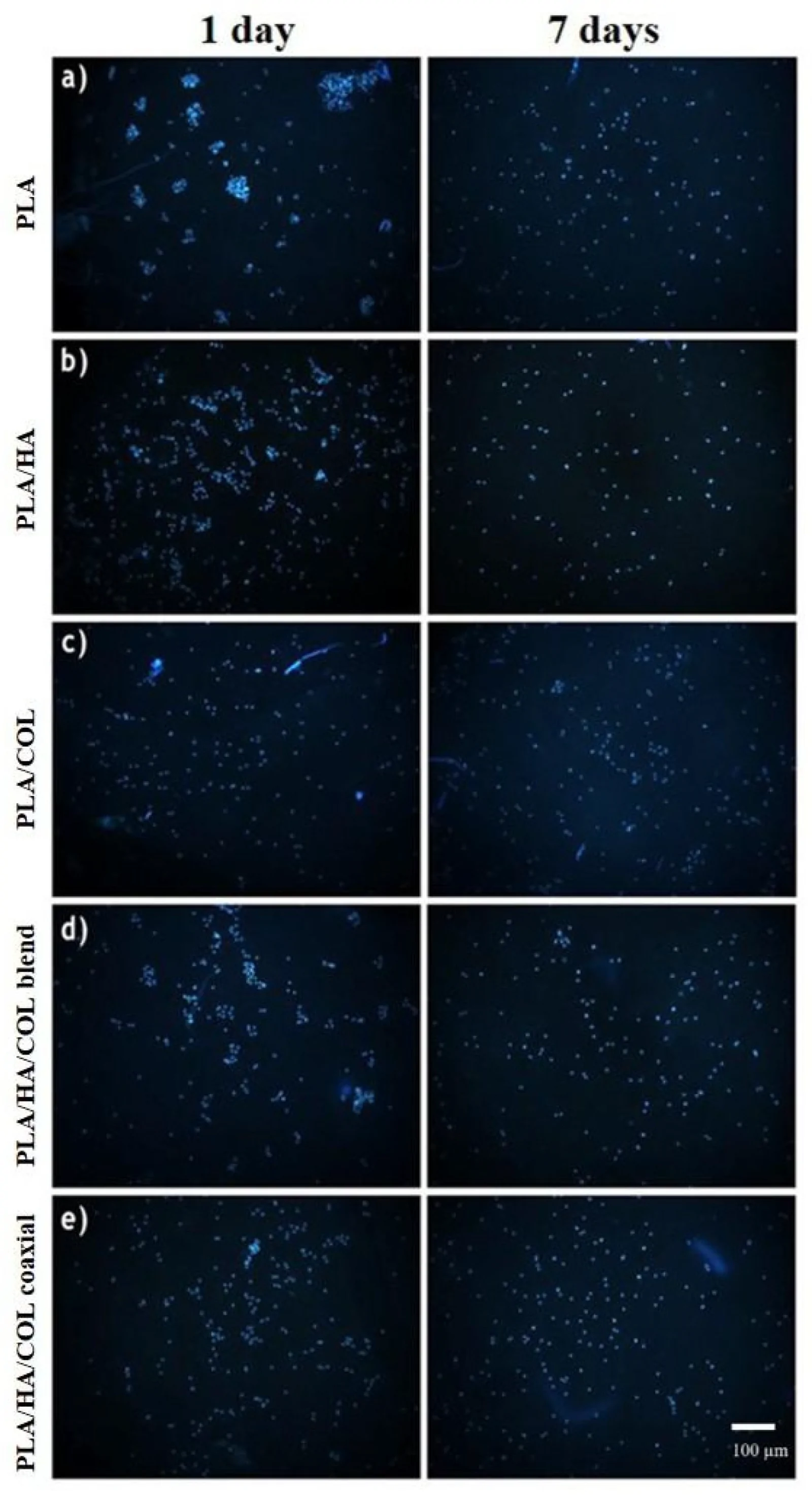
Fluorescent images of (**a**) PLA, (**b**) PLA/COL, (**c**) PLA/HA, (**d**) PLA/HA/COL blend, and (**e**) PLA/HA/COL coaxial nanofibers in hMSCs cell lines after 1 and 7 days of incubation. Morphology of hMSCs cell lines growing in 2D and 3D cell culture conditions. hMSCs cells show significant alteration in cellular morphology (20× magnification) using an inverted microscope (Scale bar = 100 µm).

**Figure 7:**
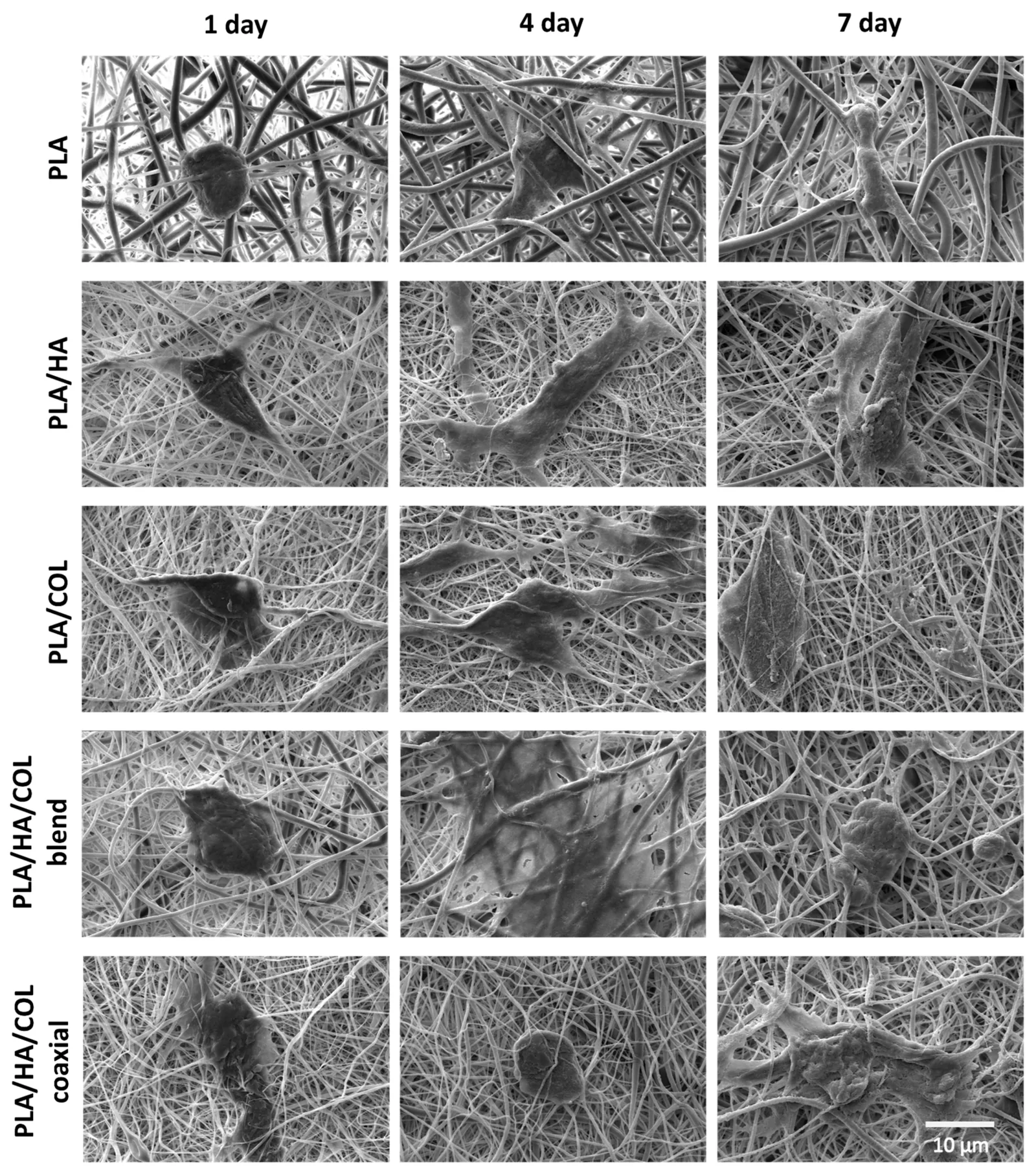
SEM images of hMSCs on PLA, PLA/HA, PLA/COL, PLA/HA/COL blend, and PLA/HA/COL coaxial nanofiber on 1st, 4th, and 7th days.

**Table 1:** Polymeric concentrations of experimental solutions.

Groups	PLA (*w*/*v*, %)	COL (*w*/*v*, %)	HA (*w*/*v*, %)
PLA	10	-	-
PLA/HA	10	-	1
PLA/COL	10	1	-
PLA/HA/COL blend	10	1	1
PLA/HA/COL coaxial (Inner layer PLA/COL Outer layer PLA/HA)	10	1	1

**Table 2:** Mechanical properties of nanofibers.

Nanofibers	Tensile Strength (MPa)	Tensile Strain (%)
PLA	2.108 ± 0.172	84.713 ± 2.984
PLA/HA	3.425 ± 0.269	14.254 ± 5.07
PLA/COL	4.94 ± 0.227	35.76 ± 1.565
PLA/HA/COL blend	3.69 ± 0.2	35.999 ± 0.647
PLA/HA/COL coaxial	4.651 ± 0.15	21.664 ± 6.564

## Data Availability

The original contributions presented in this study are included in the article. Further inquiries can be directed to the corresponding author.

## Abbreviations

The following abbreviations are used in this manuscript:

COL	Collagen
DSC	Differential scanning calorimetry
ECM	Extracellular matrix
FDA	Food and Drug Administration
FT-IR	Fourier transform infrared spectroscopy
HA	Hyaluronic acid
hMSCs	Human mesenchymal stem cells
MTT	(3-(4,5-Dimethylthiazol-2-yl)-2,5-diphenyltetrazolium bromide)
PBS	Phosphate-buffered saline
PCL	Poly-caprolactone
PLA	Polylactic acid
SEM	Scanning electron microscopy
T_g_	Glass transition temperature
T_m_	Melting temperature
TTE	Tendon tissue engineering
